# Author Correction: High throughput computations of the effective removal of liquified gases by novel perchlorate hybrid material

**DOI:** 10.1038/s41598-024-68140-0

**Published:** 2024-08-06

**Authors:** Tomsmith O. Unimuke, Hitler Louis, Onyinye J. Ikenyirimba, Gideon E. Mathias, Adedapo S. Adeyinka, Chérif Ben Nasr

**Affiliations:** 1 Computational and Bio-Simulation Research Group, University of Calabar, P.M.B 1115, Calabar, Nigeria; 2 Department of Pure and Applied Chemistry, University of Calabar, P.M.B 1115, Calabar, Nigeria; 3 Department of Chemical Sciences, Research Centre for Synthesis and Catalysis, University of Johannesburg, Johannesburg, 2006 South Africa; 4 Laboratoire de Chimie des Matériaux, Faculté des Sciences de Bizerte, Université de Carthage, 7021 Zarzouna, Tunisie

Correction to: *Scientific Reports* 10.1038/s41598-023-38091-z, published online 05 July 2023

The original version of this Article contained an error in Supplementary Figure S1. During preparation of the Supplementary Information file the FTIR spectrum of another similar compound was erroneously included.

The original Supplementary Information file is provided below.

The Supplementary Information file has been corrected.

## Supplementary Information

Supplementary Information.

