# Supplementary material for: Author Correction: High throughput computations of the effective removal of liquified gases by novel perchlorate hybrid material
Source: Sci Rep. 2024 Aug 6;14:18235. doi: 10.1038/s41598-024-68140-0 (PMC11303688; doi:10.1038/s41598-024-68140-0)
Supplement: Supplementary file 1 — Supplementary Information. [file 41598_2024_68140_MOESM1_ESM.docx]

**High throughput computations of the effective removal of liquified gases by novel perchlorate hybrid material.**

**Tomsmith O. Unimuke ^a,b*^, Hitler Louis ^ab*^, Onyinye, J. Ikenyirimba ^a^, Gideon E. Mathias ^a^, Adedapo S. Adeyinka ^c^ and Chérif Ben Nasr^d^**

^a^ Computational and Bio-Simulation Research Group, University of Calabar, Calabar, P.M.B 1115, Nigeria.

^b^Department of Pure and Applied Chemistry, University of Calabar, Calabar, P.M.B 1115, Nigeria.

^c^Research Centre for Synthesis and Catalysis, Department of Chemical sciences, University of Johannesburg 2006, South Africa

^d^Laboratoire de Chimie des Matériaux, Université de Carthage, Faculté des Sciences de Bizerte, 7021 Zarzouna, Tunisie

**^*^Corresponding author’s email**: [ojtomtsm@gmail.com](mailto:ojtomtsm@gmail.com) and [louismuzong@gmail.com](mailto:louismuzong@gmail.com)

**Table S1.** Crystallographic data and structure refinement of [C_6_H_6_Cl_2_N] ClO_4_

| Chemical formula | | | C_6_H_6_Cl_3_NO_4_ | |
| --- | --- | --- | --- | --- |
| Formula weight (g.mol^-1^) | | | 262.48 | |
| Temperature (K) | | | 183 | |
| Crystal System | | | Orthorhombic | |
| Space group | | | *Pbca* | |
| Unit cell dimensions | | | *a*= 10.4483 (8) Å  *b* = 7.6093 (7) Å  *c* = 23.558 (3) Å |  |
| *Z* | | | 8 | |
| Cell volume (Å^3^) | | | 1873.0 (3)  0.96  0.30 × 0.30 × 0.30 | |
| Absorption coefficient µ (mm^−1^) | | |  |  |
| Crystal dimensions (mm^3^) | | |  |  |
| Color, shape | | | colorless, Block | |
| Diffractometer | | Mercury CCD System (Rigaku) | | |
| *θ* range (°) | | *θ*_min_ = 3.4°,*θ*_max_ = 27.5° | | |
| Index range (h, k, l) | | *h* = −13→13, *k* = −9→8, *l* = −30→21 | | |
| No. of measured, independent and observed with *I*>2*σ*(*I*) reflections | | 12421, 2125, 1927 | | |
| *R*_int_ | | 0.039 | | |
| Absorption Correction: Integration | | *T*_min_ = 0.611, *T*_max_ = 0.749 | | |
| Radiation type | | Mo (K_α_) *λ*(Å) = 0.71075 | | |
| *R, wR*^2^ | 0.054, 0.132 | | | |
| Goodness-of-fit on *F*^2^ | 1.15 | | | |
| **_max_, **_min_ (e Å^−3^) | 0.44, −0.70 | | | |

**Table S2:** Structural parameter of the studied adsorbed gas molecules

| Selected bond lengths of PCl- adsorbed gases (Å) | | | | | | | |  |
| --- | --- | --- | --- | --- | --- | --- | --- | --- |
| Pcl@COCl_2_ | | **Pcl@CO_2_** | | **Pcl@NO_2_** | | **Pcl@SO_2_** | | **Pcl@H_2_S** |
| C_20_-O_17_ | 2.785 | C_20_-O_17_ | 2.789 | O_17_-N_22_ | 1.447 | O_17_-S_22_ | 3.198 | H_21_-H_7_ |
| C_1_-C_2_ | 1.362 | C_2_-C_3_ | 1.433 | C_1_-C_2_ | 1.359 | C_1_-C_2_ | 1.359 | C_5_-C_6_ 1.402 |
| C_3_-C_4_ | 1.439 | C_5_-C_6_ | 1.402 | C_3_-C_4_ | 1.436 | C_3_-C_4_ | 1.436 | C_3_-C_4_ 1.437 |
| C_6_-Cl_11_ | 1.705 | C_4_-Cl_10_ | 1.716 | C_6_-Cl_11_ | 1.702 | C_6_-Cl_11_ | 1.702 | C_6_-Cl_11_ 1.706 |
| N_12_-H_14_ | 1.075 | N_12_-H_14_ | 1.071 | N_12_-H_14_ | 1.054 | N_12_-H_14_ | 1.054 | N_12_-H_14_ 1.059 |
| Cl_15_-O_16_ | 1.489 | Cl_15_-O_18_ | 1.423 | Cl_15_-O_17_ | 1.431 | Cl_15_-O_17_ | 1.431 | Cl_15_-O_17_ 1.443 |
| C_20_-O_23_ | 1.739 | C_20_-O_22_ | 1.150 | N_12_-C_3_ | 1.313 | N_12_-C_3_ | 1.313 | N_12_-C_3_ 1.313 |
| C_20_-O_23_ | 1.166 | C_20_-O_21_ | 1.159 | C_4_-Cl_10_ | 1.715 | C_4_-Cl_10_ | 1.715 | H_14_-O_16_  1.598 |
| H_14_-O_16_ | 1.521 | H_14_-O_16_ | 1.532 | O_16_-H_14_ | 1.621 | O_16_-H_14_ | 1.621 | S_20_-H_22_  1.343 |
| Selected bond angles of PCl- adsorbed gases ($\boldsymbol{^{\circ}}$) | | | | | | | |  |
| Pcl@COCl_2_ | | **Pcl@CO_2_** | | **Pcl@NO_2_** | | **Pcl@SO_2_** | | **Pcl@H_2_S** |
| O_17_-C_20_-O_23_ | 99.91 | O_17_-C_20_-O_21_ | 89.529 | O_17_-N_22_-O_21_ | 110.07 | C_15_-O_17_-S_22_ | 89.07 | H7-H21-S22 |
| O_17_-C_20_-Cl_21_ | 86.45 | O_17_-C_20_-O_22_ | 92.78 | Cl_15_-O_17_-N_22_ | 116.66 | O_17_-S_22_-O_20_ | 81.68 |  |
| Cl_15_-O_17_-C_20_ | 138.08 | Cl-O_17_-C_20_ | 98.53 | O_17_-N_22_-O_20_ | 117.08 | O_17_-S_22_-O_21_ | 138.99 |  |
| O_17_-C_20_-Cl_22_ | 84.45 | O_21_-C_20_-O_22_ | 177.47 | O_20_-N_22_-O_21_ | 132.84 | O_22_-S_22_-O_21_ | 115.88 |  |
| Cl_15_-O_16_-H_14_ | 109.32 | Cl_15_-O_16_-H_14_ | 109.82 | Cl_15_-O_16_-O_18_ | 32.26 | Cl_10_-O_4_-O_3_ | 118.89 | Cl_15_-O_17_-O_18_ 33.59 |
| O_17_-Cl_15_-O_19_ | 35.26 | O_17_-Cl_15_-O_19_ | 35.36 | O_18_-Cl_15_-O_19_ | 115.91 | O_19_-Cl_15_-O_16_ | 106.56 | O_19_-Cl_15_-O_17_ 111.20 |
| O_23_-C_20_-Cl_22_ | 123.32 | C_20_-O_21_-O_22_ | 177.47 | N_22_-O_21_-O_23_ | 23.63 | N_12_-H_14_-O_16_ | 164.28 | N_12_-H_14_-O_16_ 163.05 |
| C_1_-C_2_-C_3_ | 120.35 | C_2_-C_3_-C_4_ | 118.23 | C_4_-C_5_-C_6_ | 119.32 | C_1_-C_2_-C_3_ | 120.55 | C_3_-C_4_-C_5_ 120.55 |
| Cl_10_-C_4_-C_5_ | 120.21 | Cl_11_-C_5_-C_6_ | 118.67 | Cl_11_-C_6_-C_1_ | 120.06 | Cl_10_-C_4_-C_5_ | 120.34 | Cl_10_-C_4_-C_3_ 118.92 |
| N_12_-H_13_-H_14_ | 29.13 | N_12_-H_13_-H_14_ | 119.85 | N_12_-H_13_-H_14_ | 33.27 | N_12_-H_13_-H_14_ | 120.24 | N_12_-H_13_-H_14_ 163.30 |
| N_12_-H_3_-H_4_ | 121.61 | C_3_-N_12_-H_13_ | 119.09 | C_3_-N_12_-H_14_ | 37.95 | S_22_-O_20_-O_21_ | 32.05 | H_21_-S_20_-H_22_ 92.54 |
| N_12_-C_3_-C_4_ | 118.13 | N_12_-C_3_-C_2_ | 120.14 | O_17_-N_22_-O_21_ | 110.07 | C_3_-N_12_-H_13_ | 119.83 | C_3_-N_12_-H_14_ 118.70 |

**Table S3:** Geometric properties of the studied gases.

| Gas molecule | Before adsorption | | | | After adsorption | | | |
| --- | --- | --- | --- | --- | --- | --- | --- | --- |
|  | Bond length (Å) | | Bond angle ($^{\circ}$) | | Bond length (Å) | | Bond angle ($^{\circ}$) | |
| CO_2_ | C-O | 1.150 | C-O-O | 180 | C-O | 1.160 | C-O-O | 179.95 |
| COCl_2_ | C-Cl | 1.741 | C-Cl-Cl | 180 | C-Cl | 1.755 | C-Cl-Cl | 115.89 |
| H_2_S | H-S | 1.346 | H-S-H | 92.25 | H-S | 1.337 | H-S-H | 92.55 |
| SO_2_ | S-O | 1.180 | S-O-O | 118.60 | S-O | 1.430 | S-O-O | 115.89 |
| NO_2_ | N-O | 1.180 | N-O-O | 135.04 | N-O | 1.178 | N-O-O | 132.84 |





**Figure S1:** IR Spectrum of the synthesize hybrid material

| **Table S4:** Thermodynamic properties of the studied gases | **COCl_2_** | **ClO^-4^** | **Product** |
| --- | --- | --- | --- |
| E_0_ | -1033.724003 | -1967.529219 | -3001.264907 |
| E_zpe_ | 0.010742 | 0.116153 | 0.127776 |
| H_corr_ | -1033.708407 | -1967.398842 | -3001.117047 |
| G_corr_ | -1033.741162 | -1967.457519 | -3001.191058 |
| E_o_ +E_ZpE_ | -1033.729288 | -1967.41307 | -3001.137131 |
| E_o_+E_tot_ | -2067.448006 | -3935.05844 | -6002.529814 |
| E_o_+H_corr_ | -2067.43241 | -3934.928061 | -6002.381954 |
| E_o_+G_corr_ | -2066.981192 | -3934.98874 | -6002.455965 |
| Δ_f_H^0^(298k) |  | -13.4805825kcal/mol |  |
| Δ_f_G^0^ (298k) |  | -304.9857075kcal/mol |  |
|  |  |  |  |
|  | **CO_2_** | **ClO^-4^** | **Product** |
| E_0_ | -188.585732 | -1967.529219 | -2156.128321 |
| E_zpe_ | 0.012003 | 0.116153 | 0.129237 |
| H_corr_ | -188.570192 | -1967.398842 | -2155.980898 |
| G_corr_ | -188.595055 | -1967.457519 | -2172.520303 |
| E_o_ +E_ZpE_ | -188.573729 | -1967.41307 | -2155.999084 |
| E_o_+E_tot_ | -377.171464 | -3935.05844 | -4312.256642 |
| E_o_+H_corr_ | -377.155924 | -3934.928061 | -4312.109219 |
| E_o_+G_corr_ | -377.180787 | -3934.98874 | -4328.648624 |
| Δ_f_H^0^(298k) |  | -2705832.701kcal/mol |  |
| Δ_f_G^0^ (298k) |  | -2705886.378kcal/mol |  |
|  |  |  |  |
|  | **NO_2_** | **ClO^-4^** | **Product** |
| E_0_ | -205.065273 | -1967.529219 | -2172.600493 |
| E_zpe_ | 0.009192 | 0.116153 | 0.130139 |
| H_corr_ | -205.052214 | -1967.398842 | -2172.452293 |
| G_corr_ | -205.080044 | -1967.457519 | -2172.520303 |
| E_o_ +E_ZpE_ | -205.056081 | -1967.41307 | -2172.470354 |
| E_o_+E_tot_ | -410.130546 | -3935.05844 | -4345.200986 |
| E_o_+H_corr_ | -410.117487 | -3934.928061 | -4345.052786 |
| E_o_+G_corr_ | -410.145317 | -3934.98874 | -4345.120796 |
| Δ_f_H^0^(298k) |  | -4.541845 kcal/mol |  |
| Δ_f_G^0^ (298k) |  | 8.3212775Kcal/mol |  |
|  |  |  |  |
|  | **SO_2_** | **ClO^-4^** | **Product** |
| E_0_ | -548.614005 | -1967.529219 | -2516.161982 |
| E_zpe_ | 0.007384 | 0.116153 | 0.125483 |
| H_corr_ | -548.602631 | -1967.398842 | -2516.017691 |
| G_corr_ | -548.631414 | -1967.457519 | -2516.088247 |
| E_o_ +E_ZpE_ | -548.606621 | -1967.41307 | -2516.036499 |
| E_o_+E_tot_ | -1097.22801 | -3935.05844 | -5032.323964 |
| E_o_+H_corr_ | -1097.216636 | -3934.928061 | -5032.179673 |
| E_o_+G_corr_ | -1097.245419 | -3934.98874 | -5032.250229 |
| Δ_f_H^0^(298k) |  | -21.94744 kcal/mol |  |
| Δ_f_G^0^ (298k) |  | -10.083925 kcal/mol |  |

**Table S5:** Thermodynamic properties of PClH material

|  | **ClO4** | **Aromatic fragment** | **PClH** |
| --- | --- | --- | --- |
| E_0_ | -1203.667838 | -760.670802 | -1967.529219 |
| E_zpe_ | 0.103586 | 0.011723 | 0.116153 |
| H_corr_ | -1203.556824 | -1203.555880 | -1967.398842 |
| G_corr_ | -1203.59821 | -760.687045 | -1967.457519 |
| E_o_ +E_ZpE_ | -1203.5643 | -760.65908 | -1967.41307 |
| E_o_+E_tot_ | -2407.335676 | 1521.341604 | -3935.05844 |
| E_o_+H_corr_ | -2407.335676 | -1521.3578 | -3934.928061 |
| E_o_+G_corr_ | -3611.003514 | -1520.357847 | -3934.98874 |
| Δ_f_H^0^(298k) |  | -3915.967088 kcal/mol |  |
| Δ_f_G^0^ (298k) |  | 750723.8197 kcal/mol |  |


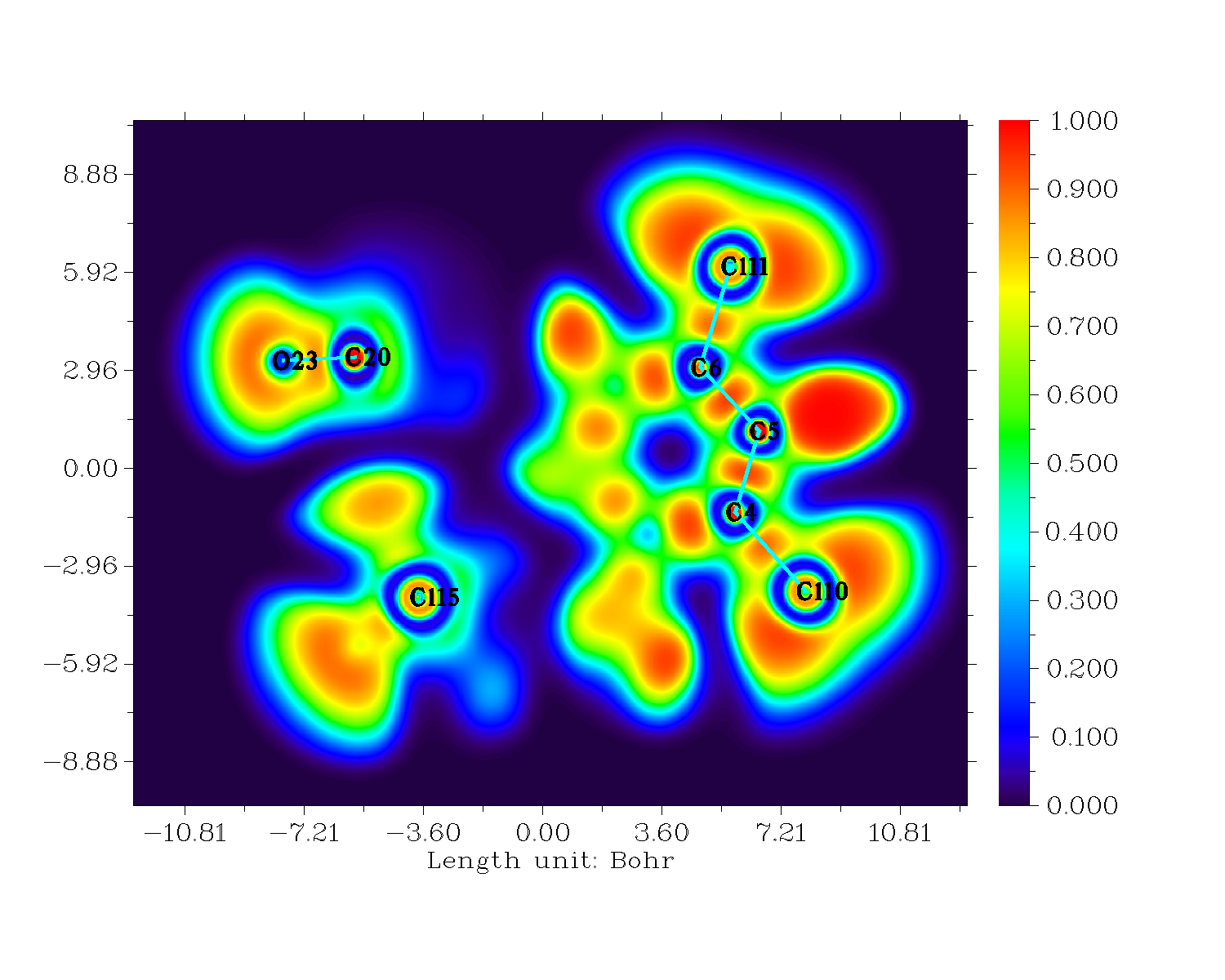


**COCl_2_@PClH**

**FigureS2:** Electron localization function maps of the studied compounds


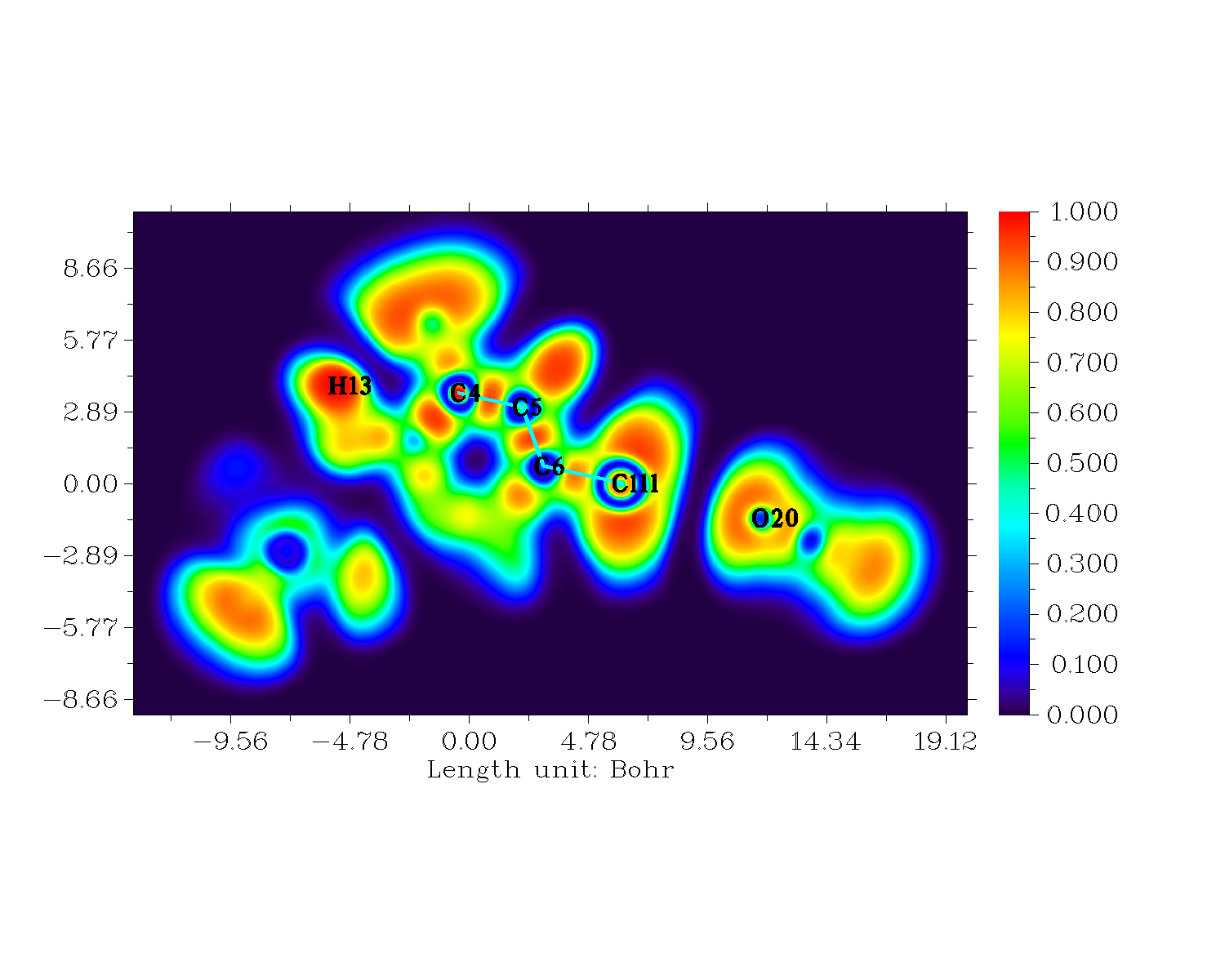


**CO_2_@PClH**


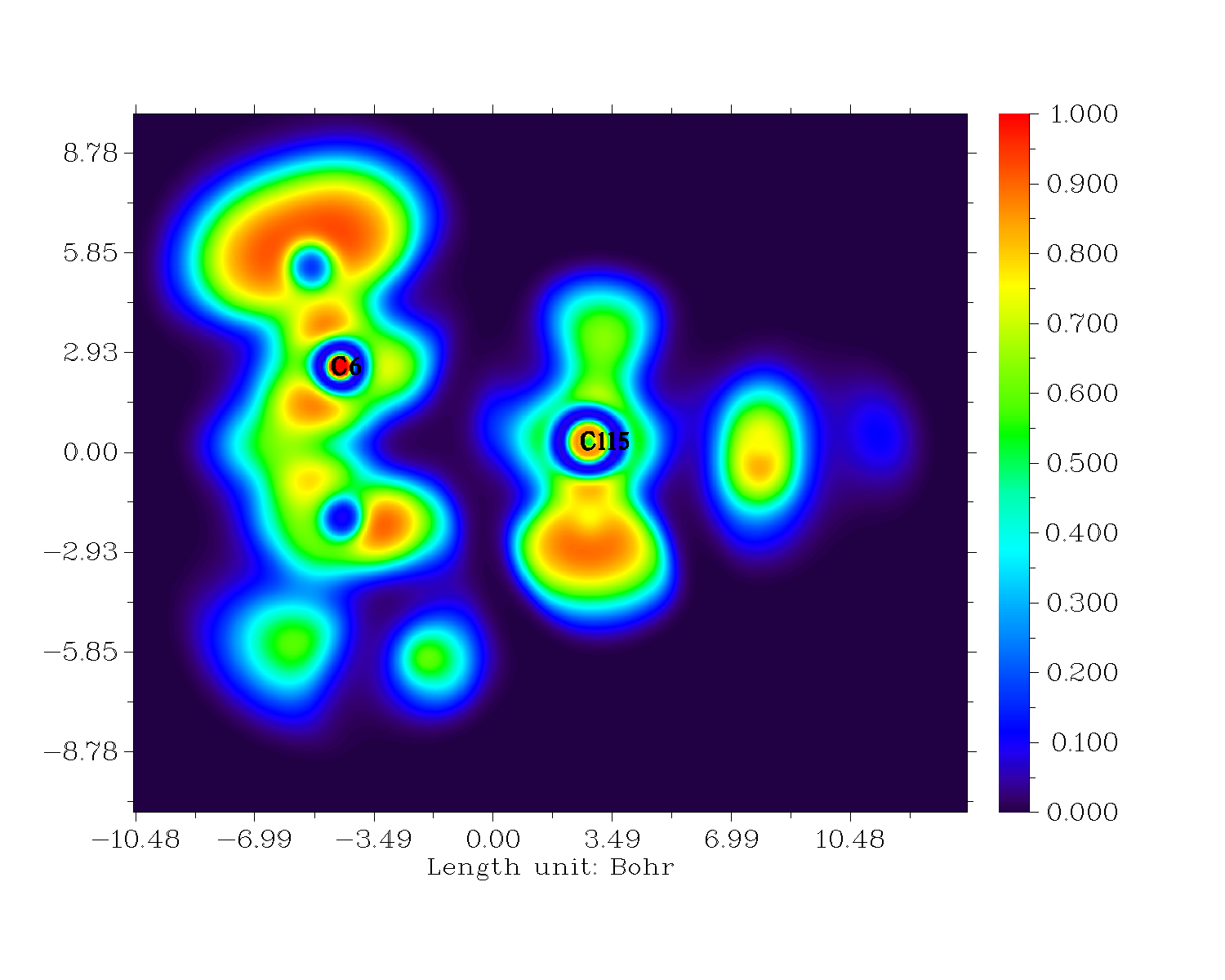


**NO_2_@PClH**


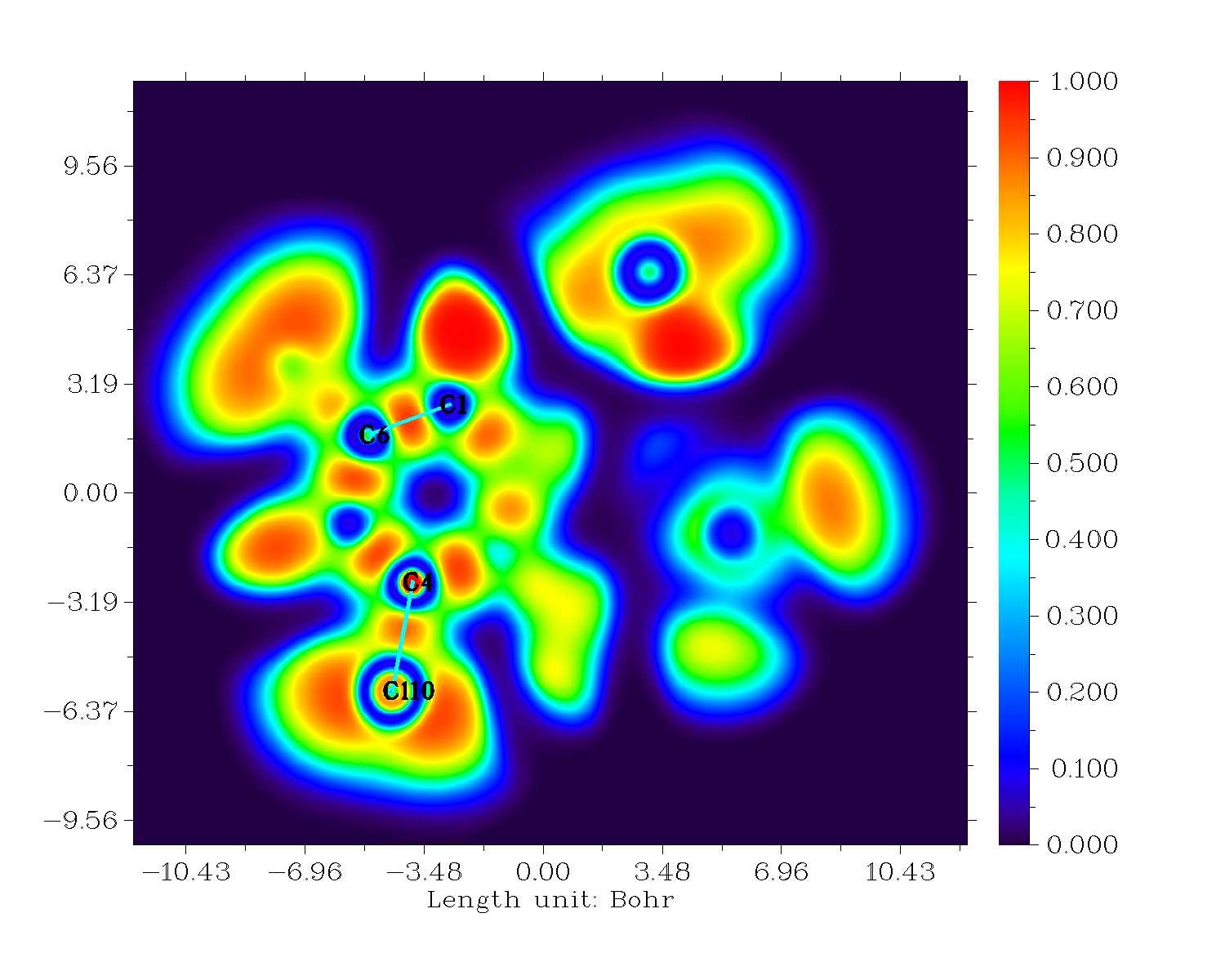


**H_2_S@PClH**


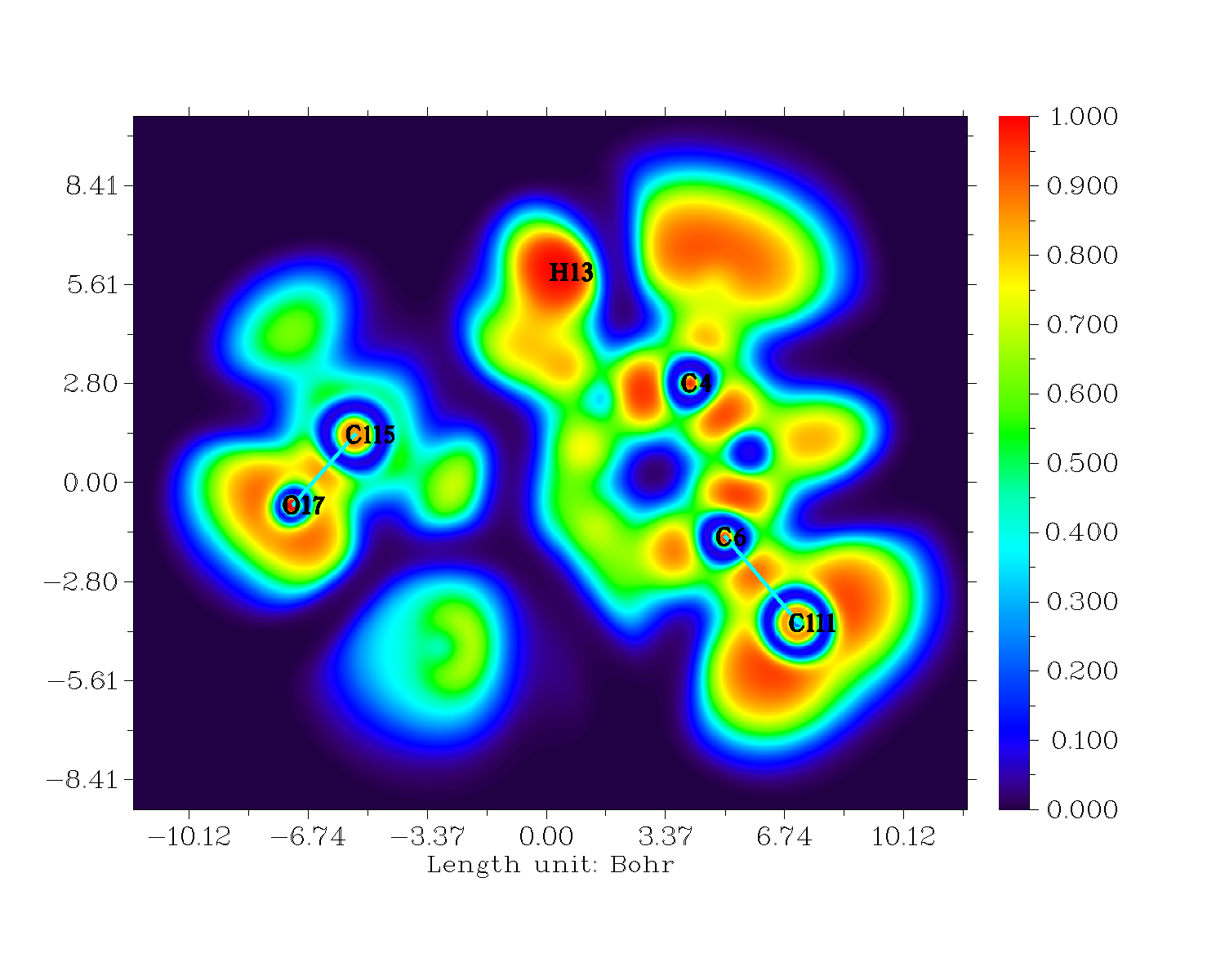


**SO_2_@PClH**
